# Impact of personality traits on learners’ navigational behavior patterns in an online course: a lag sequential analysis approach

**DOI:** 10.3389/fpsyg.2023.1071985

**Published:** 2023-05-23

**Authors:** Ahmed Tlili, Tianyue Sun, Mouna Denden, Kinshuk, Sabine Graf, Cheng Fei, Huanhuan Wang

**Affiliations:** ^1^Smart Learning Institute of Beijing Normal University, Beijing, China; ^2^Department of Health and Behavior Studies, Teachers College, Columbia University, New York, NY, United States; ^3^University Polytechnique Hauts-de-France, LAMIH, CNRS, UMR 8201, Valenciennes, France; ^4^INSA Hauts-de-France, Valenciennes, France; ^5^University of North Texas, Denton, TX, United States; ^6^School of Computing and Information Systems, Athabasca University, Edmonton, AB, Canada

**Keywords:** personality, online learning, navigational behaviors, adaptive systems, distance education, lag sequential analysis

## Abstract

Personality is considered as the internal factor that defines a person’s behavior. Therefore, providing adaptive features and personalized support in online learning by considering learners’ personalities can improve their learning experiences and outcomes. In this context, several research studies have investigated the impact of personality differences in online learning. However, little is known about how personality differences affect learners’ behavior while learning. To fill this gap, this study applies a lag sequential analysis (LSA) approach to understand learners’ navigational behavior patterns in an online three-months course of 65 learners based on their personalities. In this context, the five factor model (FFM) model was used to identify learners’ personalities. The findings revealed that learners with different personalities use different strategies to learn and navigate within the course. For instance, learners high in extraversion tend to be extrinsically motivated. They therefore significantly navigated between viewing the course module and their personal achievements. The findings of this study can contribute to the adaptive learning field by providing insights about which personalization features can help learners with different personalities. The findings can also contribute to the field of automatic modeling of personality by providing information about differences in navigational behavior based on learners’ personalities.

## 1. Introduction

Taking into consideration learners’ individual differences in computer-based learning is important as these differences can define how a given learner behave in a learning environment (Tlili et al., 2016). For instance, learners’ individual differences can affect the learning process, where some learners might find it easy to learn a particular course, whereas others find the same course difficult (Jonassen and Grabowski, 1993). Personality is widely identified as an important indicator of individual differences (Irani et al., 2003; Essalmi et al., 2017). It can affect several important predictors of academic performance, such as learning approaches, effective learning and self-regulation strategies, cognitive abilities, and academic motivation (Barrick and Mount, 1996; Diseth, 2003; Zhang, 2003; Bidjerano and Yun Dai, 2007; Clark and Schroth, 2010; Swanberg and Martinsen, 2010).

In a comprehensive review, Tlili et al. (2016) highlighted the importance of understanding learners’ behaviors based on their personalities to provide adaptive computer-based learning experiences accordingly. Fatahi (2019) also reported that adaptive e-learning environments based on personality improved learners’ performance. Lai et al. (2019) further emphasized the importance of adaptive e-learning based on learners’ personality traits, which facilitated learning efficiency and met learners’ demands; thus, learners may understand the learning materials better. Denden et al. (2021) found that different personality traits prefer different game elements in gamification. They therefore recommended providing adaptive design of gamified online learning systems based on learners’ personality traits. It is therefore important to investigate how learners with different personalities use and navigate through online courses. Navigational behavior refers to how learners navigate through the course and in which order they visit different kinds of learning objects and activities (Graf et al., 2010). Adaptive navigation support, in terms of recommending learners a suitable way through learning materials and activities, is one of the two main ways for adding adaptive functionality to learning systems (Brusilovsky, 2001). Hence, learning about how learners with different personalities navigate through an online course can help to provide adaptive learning features and provide personalized support, which enhance their learning experiences and outcomes.

While there is much agreement on the effects of personality on learning, limited empirical findings are found related to leaners’ navigational behavior patterns in online courses based on their personalities. Therefore, to fill this gap, this study analyzes the learners’ log files on the learning management system Moodle using lag sequential analysis (LSA) to identify their navigational behavior patterns based on their personalities. LSA allows conducting in-depth investigation on learning behaviors or event chains that occur at frequencies greater than chance (Sackett, 1978). In education specifically, LSA takes transitional relationships into consideration to identify temporal differences in learning behaviors (Chen et al., 2017). For example, Graf et al. (2010) applied LSA to investigate the impact of learners’ learning styles on their navigational behaviors in an online course. Cheng et al. (2019) applied LSA to explore the process of co-construction of knowledge where 24 sixth-grade learners are competing in an augmented reality mathematic game. Tlili et al. (2021) also applied LSA to examine the behavioral pattern differences among learners from either China, Tunisia or Serbia who enrolled in an online six-week course. Wang et al. (2022) further applied LSA to investigate how gender might moderate learners’ online learning behavioral patterns.

To identify the learners’ personalities, this study relies on the five-factor model (FFM), which is one of the most common psychological models (Franić et al., 2014) and is frequently used in education (Tlili et al., 2016). It attributes five personality dimensions, namely openness, conscientiousness, extraversion, agreeableness, neuroticism, often abbreviated as OCEAN. Each of these dimensions is discussed in the next section.

## 2. Theoretical background

Online learning differs from traditional face-to-face learning in the way that it does not require learners to present themselves in an actual classroom setting (Wang et al., 2013). Learners who enroll in online courses have greater flexibility in their learning process as they decide when, where and how to navigate the learning materials (Wang et al., 2013; Hong et al., 2021). With the rapid evolution of technology, online learning research has gained an increasing attention as through technology it can enhance learners’ learning engagement and achievement (Wengrowicz et al., 2018). In this context, several studies called for more investigation on the factors that could affect the process of online learning, such as personality (Sun et al., 2020; Hong et al., 2021).

### 2.1. Personality

Personality is defined as the internal factor that makes a person’s behavior consistent over time (Child, 1968). It accounts for the individual differences in emotional, interpersonal, motivational and other aspects (McCrae and John, 1992; Gustavsson et al., 2003). Numerous personality theories and models exist in the literature, such as five factor model (FFM; McCrae and John, 1992), Myer Briggs types (Myers et al., 1998) and Han Eysenck’s model (Boeree, 2006).

This study uses the Five Factor Model (FFM) of personality, which is one of the most accepted personality models in the literature (Trull and Sher, 1994; Barrick et al., 2003; Costa and McCrae, 2009) to describe learners’ personality traits. It is validated across various countries and cultures (John et al., 2008; Gurven et al., 2013; Novikova and Vorobyeva, 2019; Murphy et al., 2021). FFM is derived from common language descriptors (DeYoung et al., 2007; Ackerman, 2020). It is an accurate personality model and it is easy to be reused in different contexts (DeYoung et al., 2007). FFM consists of five personality dimensions, namely (John and Srivastava, 1999): (1) extraversion focuses on a person’s sociability, activeness and enthusiasm; (2) agreeableness emphasizes a person’s compliance, altruism and generosity; (3) conscientiousness relates to a person’s self-discipline, achievement-striving and responsibility; (4) neuroticism is concerned with a person’s emotional stability, hostility and impulsivity; and (5) openness refers to a person’s interest in new experience, curiosity and imagination.

### 2.2. Effects of personality on online learning

Personality has been proved essential to create an adaptive online learning environment. Many studies have emphasized the importance of providing adaptive computer-assisted learning environments based on personality, as personality affects individual learning preferences and learning processes (Tlili et al., 2016; Fatahi, 2019; Lai et al., 2019). For instance, Fatahi (2019) designed an adaptive online learning environment by gathering introverts’ learning preferences. The adaptive e-learning system helped learners perform better with a higher grade. Harrington and Loffredo (2010) claimed that compared to learners high in extraversion who preferred the traditional in-person learning, learners low in extraversion found online learning more comfortable since they did not need to do face-to-face communication with their peers. In terms of online learning adoption, while agreeableness predicted the lowest adoption value, openness and conscientiousness were positively correlated with online learning adoption (Haron and Sahar, 2010; Harrington and Loffredo, 2010).

Furthermore, Arockiam and Selvaraj (2013) proved that personality plays an important role in learners’ preferences of the design of online learning interfaces. Specifically, learners high in extraversion found it easier to recall information colored in blue with “Times” font style, whereas learners high in neuroticism found it easier to recall information colored in green with “Times” font style. Personality can also mediate the learning process (Al-Dujaily et al., 2013). For instance, learners high in extraversion were prone to critical thinking learning approaches in online learning environments (Zhang, 2003); learners high in neuroticism preferred highly structured learning environments (Furnham, 1992); and learners high in conscientiousness preferred organizing learning approaches and advanced time management (Moldasheva and Mahmood, 2014). Furthermore, learners with diverse personality types significantly engaged in learning activities differently. For instance, Lee and Lee (2006) found that compared to learners low in extraversion, learners high in extraversion were more social and interactive. They, therefore, posted more messages in the web-based discussion forums. Yu (2021) found that learners high in agreeableness, conscientiousness, and openness personality traits outperformed those high in extraversion and neuroticism personality traits in online learning outcomes during the covid-19. Finally, Denden et al. (2018) and Tlili et al. (2019) relied on the FFM personality model and revealed that learners’ personalities affect the way learners engage in different learning environments, including educational games and Moodle.

### 2.3. Research gap and the purpose of the study

Research on online learning effectiveness has experienced a shift towards focusing on learner characteristics or differences like personality traits (Chai et al., 2022). However, only a few studies focused on the relationship between personality and online learning behaviors, and these studies focused on analyzing single behaviors, such as note taking or discussing course related topics with peers (Lee and Lee, 2006; Wu and Hou, 2015). Shang et al. (2020) mentioned that such single behaviors cannot reflect the learners’ cognitive engagement and learning behaviors characteristics in details. Yang et al. (2016) further mentioned that investigating the behavior transformation sequence can deeply explain how learners engaged in a given course and their cognitive behaviors. As such, it would be important to do further studies that consider more complex behaviors and investigate their relationship to personality traits.

Additionally, the existing studies aimed at drawing connections between personality and online learning behaviors focusing on a specific personality trait, such as procrastination (Hong et al., 2021). To the best of our knowledge, no research has examined the effects of a personality model, such as the five-factor model (with five personality traits), on navigational behavior patterns in an online course.

To cover this gap, this study complements the available body of research by analyzing the learners’ personality traits based on the FFM model (considering its five dimensions: extraversion, conscientiousness, agreeableness, neuroticism and openness) and their navigational behavior patterns in an online course. Specifically, this study answers the following research question: *How do personality traits of the Five Factor Model affect navigation behavioral patterns of learners in an online course?* To answer this research question, this study applies lag sequential analysis (LSA) to investigate the impact of learners’ personality on their navigational behavior patterns in a three-months online course in a public university. LSA was used in this study because it can reveal knowledge-construction behaviors’ temporal dynamics (Zhang et al., 2017; Sun et al., 2021). It estimates the probability of a given behavior to occur, as well as its successive behavior (Bakeman and Gottman, 1997). This can help researchers examine behavior patterns (e.g., Yang et al., 2015; Kucuk and Sisman, 2017; Wang et al., 2022) and understand how a given user might behave in a given context. Therefore, LSA was used in this study to understand how learners with different personalities might behave in an online course.

## 3. Method

### 3.1. Study context

Data from a three-months (the length of the semester) Basic Software (BS) course was used in this study. The course aims to help learners learn computer architecture and compilation, operating systems, and assembly language. It was chosen because it is part of the Computer Science curriculum at a public Tunisian university, and it was taught online. All the learners who participated in this experiment were already enrolled in the course. The online course system was run on the learning management system (LMS) Moodle, a free and open-source system.

**Figure 1:**
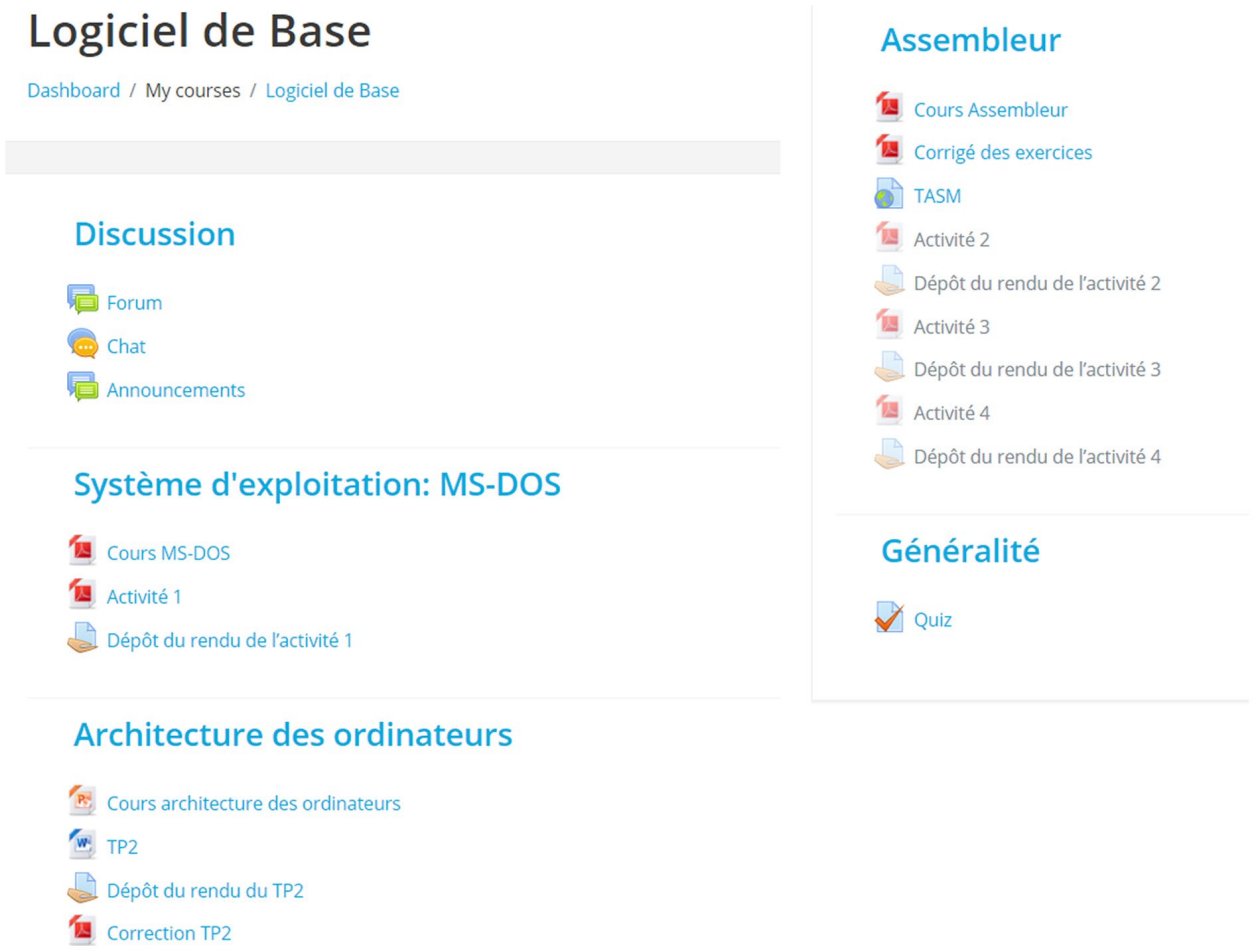
Learning activities on Moodle.

Weekly learning materials in various forms, such as videos, texts, PowerPoint presentations, external links for online resources and mental break items (e.g., pictures) were uploaded by the teacher. For each course module, the learners had to read different learning materials uploaded by the teacher, as well as finish different assignments. These assignments are quizzes to be answered on Moodle or also a particular exercise that learners had to finish on a separate Word document, and then upload it in Moodle. They also had the chance to update their uploaded assignments, if needed (before the given deadline). The learners were rewarded with a digital badge for each completed course module. Additionally, they could freely use the course forum to post their questions and to communicate with their peers (i.e., not mandatory task). The teacher was more as a facilitator by grading and providing written feedback on the uploaded assignments online. She also encouraged and helped the learners by answering questions and joining their online forum discussions, when needed. Figure 1 presents the whole learning activities on Moodle.

**Figure 2:**
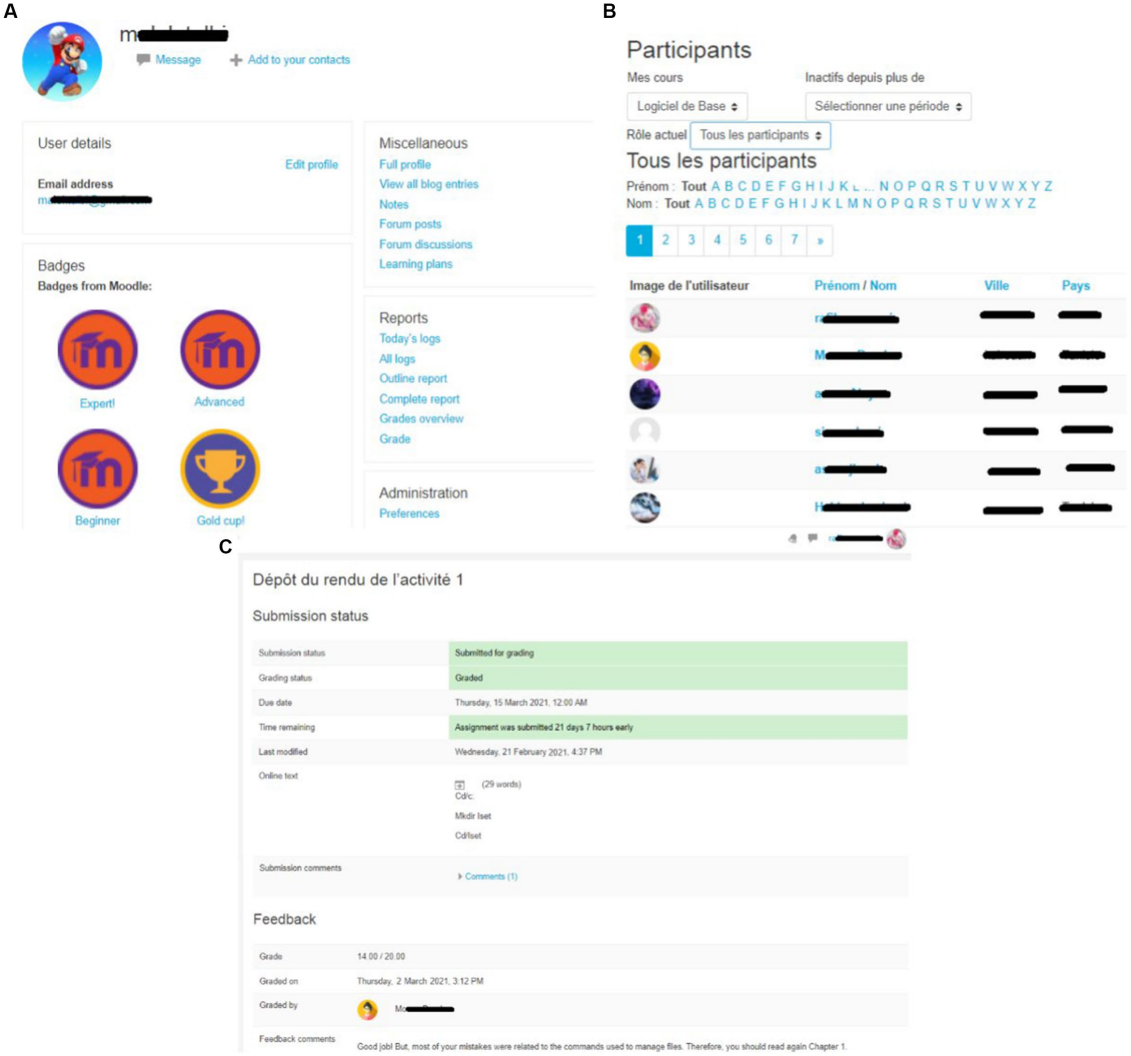
The Moodle system: **(A)** learner profile with a summary of their course achievements, **(B)** list of peers with the access of their profiles, and **(C)** examples of an assignment status.

A summary of each learner’s course achievements was displayed on their profiles. Learners had also the possibility to see their detailed course achievement report (grades, completion rate, collected badges, etc.), as well as the course achievements of their peers. For instance, as shown in Figure 2A, the learners’ profiles show a summary of their course achievements, such as their collected badges, which is visible to all learners. Additionally, the learners had the possibility to see the list of their course peers, and view their profiles (see Figure 2B). Finally, the learners can see the status of their assignments (grades or feedback given by the teacher) (see Figure 2C).

### 3.2. Participants and instrument

Participants were 92 undergraduate learners (66% of them are males and 34% are females) majoring in computer science and aged between 18 and 23. At the beginning of the semester, the learners’ personality traits were identified using the big five inventory (BFI). BFI is validated and widely used in the literature to identify individuals’ personalities (John and Srivastava, 1999). It is a five-point Likert-type questionnaire, with answers ranging from 1 (strongly disagree) to 5 (strongly agree). It consists of 44 items which cover the five personality dimensions in the FFM, such as “I am someone who is helpful and unselfish with others” for the agreeableness dimension and “I am someone who is talkative” for the extraversion dimension.

At the end of the semester (after three months), the learners’ log data was collected. To ensure an accurate analysis and findings with more representative behaviors, learners who dropped-out from the course (*n* = 27) were excluded. Therefore, the study had 65 participants. Since there is no guidance in the BFI scoring for determining whether an individual has a high or low personality trait (e.g., high extraversion or low extraversion) (Codish and Ravid, 2014), the standard *z*-score was computed, as suggested in several studies (Bidjerano and Yun Dai, 2007; Codish and Ravid, 2014). It provides information on how far a data point is from the mean. In this context, learners with *z* > 0 were considered as having a high value on the respective personality dimension (e.g., high in extraversion, high in openness, etc.), while learners with *z* < 0 were considered to have a low value on the respective personality dimension (e.g., low in extraversion, low in openness, etc.). In this present study, no learners were found with *z* = 0. Table 1 presents the mean and standard deviation of each personality dimension. Since the agreeableness personality dimension had an unbalanced number of learners (see Table 1), it was excluded from this study. Therefore, this study investigated the learners’ navigational behavior patterns of the remaining four personality traits, namely extraversion, openness, neuroticism and conscientiousness.

**Table 1:** Distribution of personality traits.

Personality dimensions	Extraversion *α* = 0.88		Agreeableness *α* = 0.82		Conscientiousness *α* = 0.84		Neuroticism *α* = 0.78		Openness *α* = 0.86	
Level	High	Low	High	Low	High	Low	High	Low	High	Low
Number of learners	35	30	46	19	31	34	31	34	35	30
Mean	3.75	2.79	4.27	3.37	4.17	3.18	3.22	2.06	4.05	3.34
SD	0.37	0.38	0.22	0.45	0.31	0.41	0.51	0.44	0.27	0.22

In our study, for each personality trait, the reliability, mean and standard deviation were calculated. As shown in Table 1, the results yielded an alpha of 0.7 or higher, which means that all the personality traits produced acceptable reliabilities.

### 3.3. Data coding and analysis

The learners’ navigational behaviors were automatically captured and stored by Moodle online. Specifically, after data cleaning, this study collected 15,869 log data from the 65 learners. These log data described 12 online learning behaviors (see Table 2), which are considered significant to the representation of learners’ navigational behaviors on learning management systems (Wang, 2017; Tlili et al., 2019). The frequency distribution of each of these online learning behaviors according to the four personality dimensions is presented in Appendix.

**Table 2:** Coding of the learning behaviors.

Learning behaviors	Learning activity	Description	Code
Course activity (CA)	Course module viewed	A learner has viewed a particular course module	CA1
	Course module completion	A learner has completed a particular course module	CA2
Assignment submission (AS)	Assignment form viewed	A learner has viewed a particular submission form (i.e., assignment, deadline, and time line)	AS1
	Assignment uploaded	A learner has uploaded a particular assignment	AS2
	Assignment updated	A learner has updated a particular assignment	AS3
	Assignment status viewed	A learner has viewed the status of a particular assignment (i.e., grades or feedback given by the teacher)	AS4
Discussion (D)	Discussion viewed	A learner has viewed the discussion on the forum	D1
	Discussion made	A learner has been involved in the discussion on the forum (i.e., write a post or reply to a discussion)	D2
Achievement result (AR)	Personal achievement viewed	A learner has viewed his/her personal course achievements	AR1
	Peers’ achievement viewed	A learner has viewed his/her peers’ course achievements	AR2
Peers (P)	The list of peers viewed	A learner has viewed the list of his/her peers taking the course	P1
	Peers’ profile viewed	A learner has viewed a specific profile of his/her peers	P2

To identify the navigational behavior patterns of each personality trait based on the learning behaviors described above, LSA was applied using GSEQ version 5.1 software (Bakeman and Quera, 2011). The motivation behind using LSA in this study is because it is a common statistical technique in behavioral science and well situated for analyzing the interaction data collected through log files (Pohl et al., 2016). LSA has been widely used to examine the behavioral transitions which happen at frequencies greater than chance (Sackett, 1978). Subsequently, a series of behavioral transitions or navigational patterns (i.e., the order in which learners go through different activities) could be identified (Graf et al., 2010).

To conduct LSA, learners’ behaviors were coded in the chronological order of their occurrences. For example, after logging into the system, a learner viewed a course module (CA1), uploaded an assignment (AS2), and then saw her achievements (AR1); this series of behaviors was thus coded as “CA1 AS2 AR1.” The *z*-score value of each connection between each sequence was calculated to determine if that connection reached the statistical significance. Bakeman and Quera (1995) stated that a *z*-score greater than 1.96 indicates that a specific sequence has reached the level of significance (*p* < 0.05). Wampold (1992) further mentioned that, as cited in McComas et al. (2009), a *z*-score does not indicate the degree to which a pattern is present. Based on this, this study therefore used the *z*-score in conjunction with a strength of association measurement, specifically Yule’s Q (Pohl et al., 2016). Yule’s Q is a transformation of the odds ratio to a [−1 … +1] range. Therefore, in this study, a transition from one code (behavior) to another was then only considered significant if the *z*-score was above the 1.96 level (the critical value assuming a normal distribution and a significance level of 0.05) and the *Q*-value was at least 0.30 (a moderate association).

The transitional probabilities, which is the conditional probability of a transition type (Cheng et al., 2017), were also calculated using GSEQ version 5.1. A transitional probability indicates the likelihood that an initial behavior follows a subsequent or same behavior.

Furthermore, prior to conducting LSA, a Pearson chi-square test was also conducted on the behavior frequency table of all the learners to determine if a significant dependence between rows and columns exist. Rows contain the initial behaviors, while columns contain the successive behaviors after conducting the initial ones.

## 4. Results and discussion

**Figure 3:**
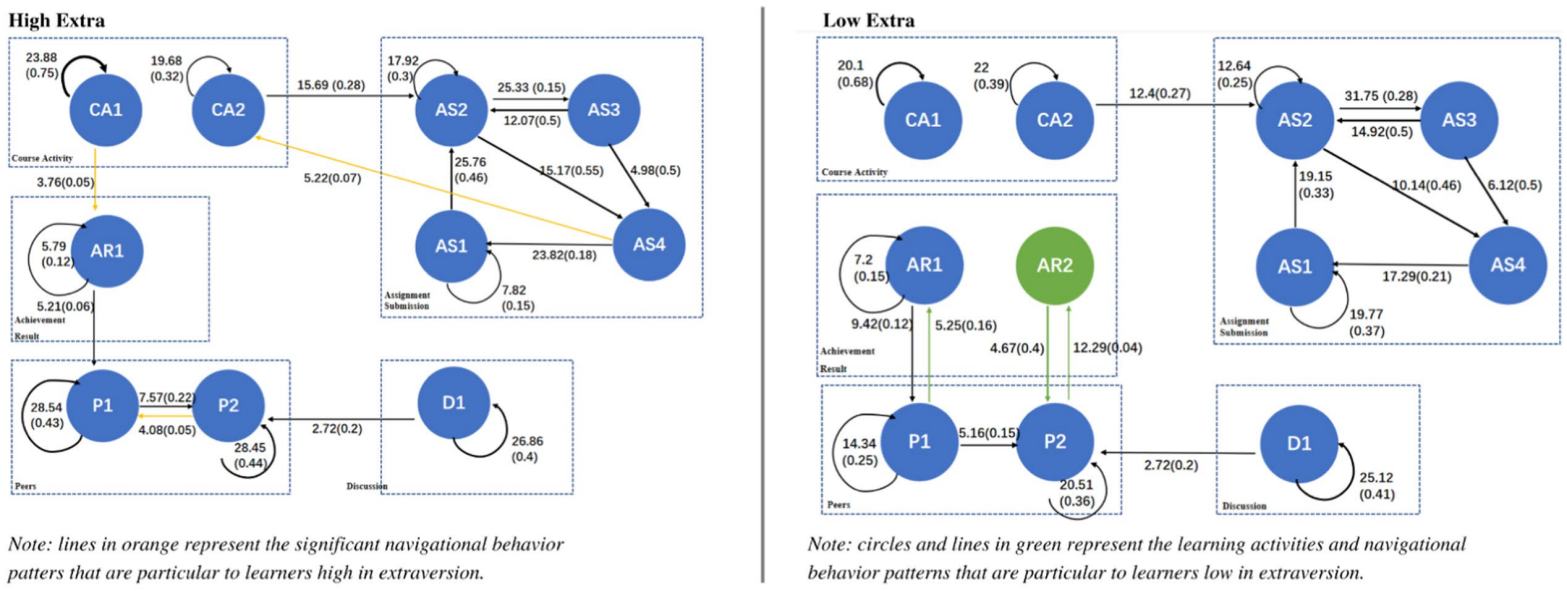
Navigational behavior patterns of learners high and low in extraversion.

**Figure 4:**
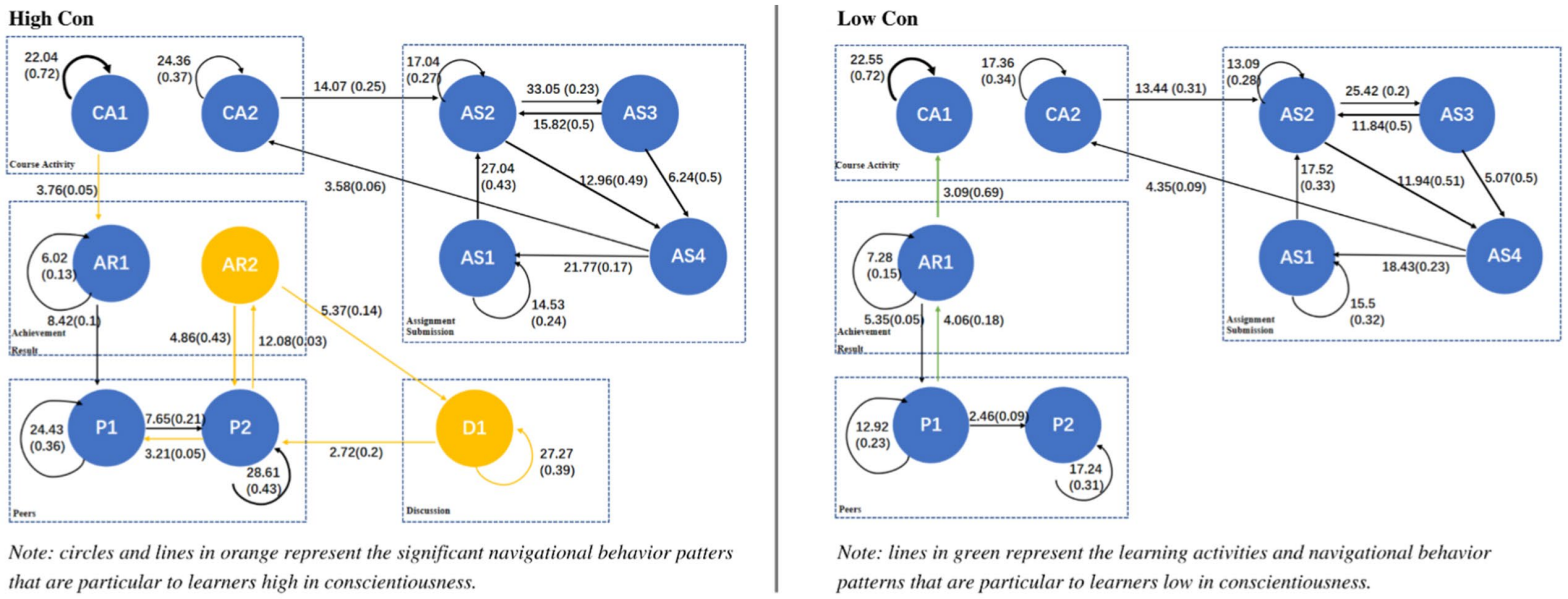
Navigational behavior patterns of learners high and low in conscientiousness.

**Figure 5:**
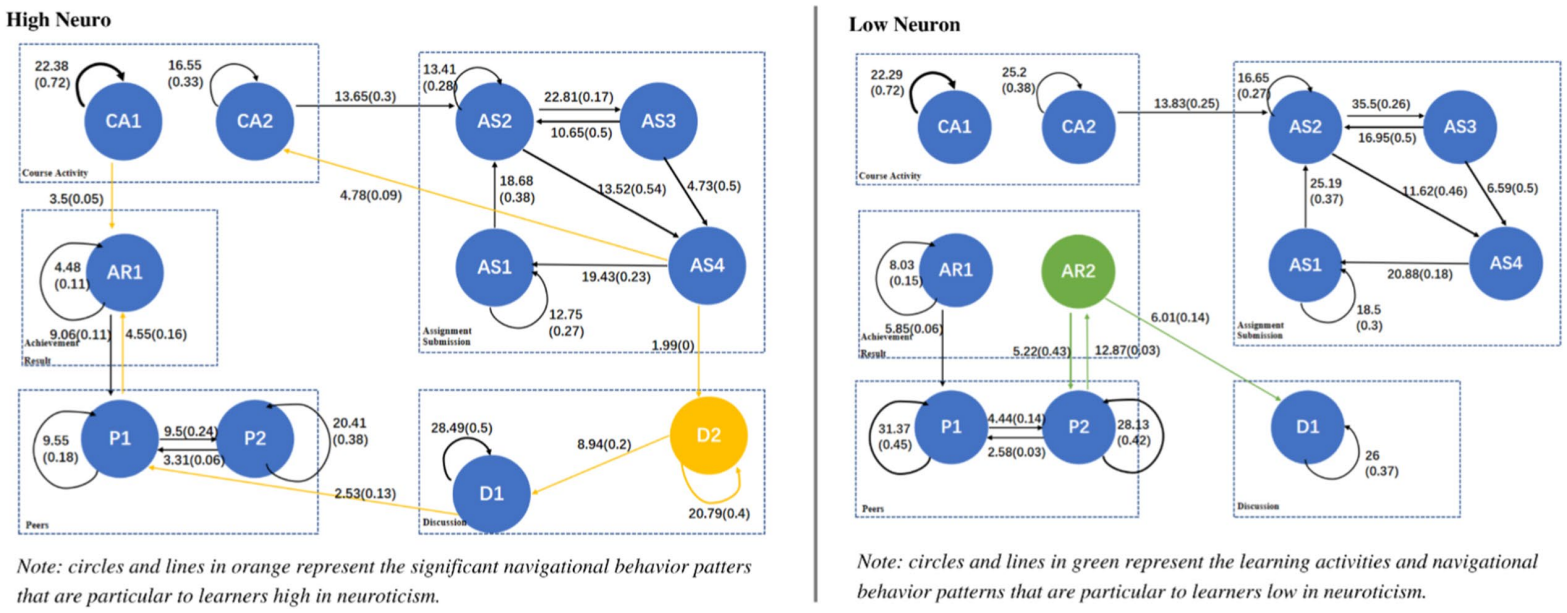
Navigational behavior patterns of learners high and low in neuroticism.

**Figure 6:**
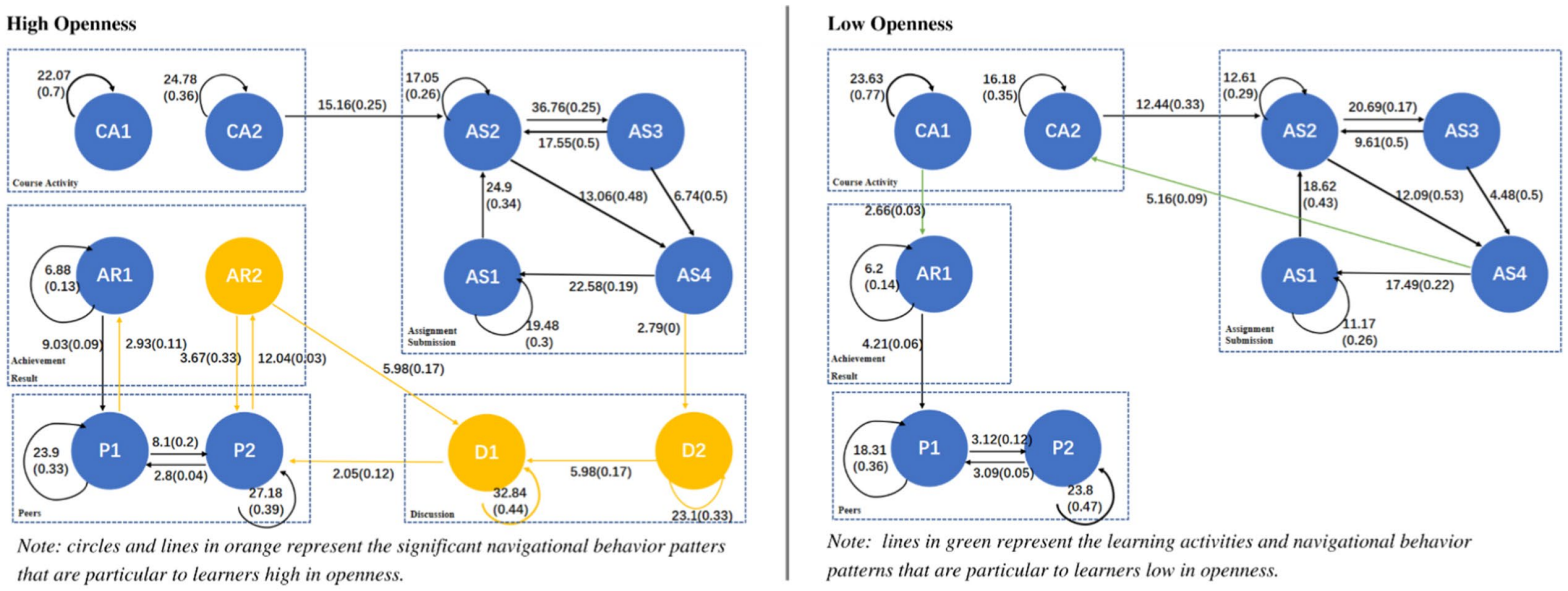
Navigational behavior patterns of learners high and low in openness.

A behavior transition diagram was drawn for each personality trait (low and high), as shown in Figures 3–6, showing those sequences which reached a significant effect. Each transition in Figures 3–6 has both significance and probability values represented on each line as follow: *Significance (Probability)*. The effect size was highlighted based on the probability of each transition, where the thicker the lines, the higher the probability of each transition. Orange and green colors were used to highlight the unique navigational behavior patterns of learners with high and low levels in each personality trait, respectively. Black color shows that the pattern was significant for low and high levels of the respective personality trait.

### 4.1. Extraversion

The obtained chi-square test results confirmed that there is a significant relation between the rows and columns of the tallied frequencies (*χ*^2^ = 7224.71, *df* = 121, *p* < 0.001 for learners high in extraversion; *χ*^2^ = 5726.87, *df* = 121, *p* < 0.001 for learners low in extraversion). Figure 3 shows the navigational behavior patterns of learners high and low in extraversion.

As shown in Figure 3, learners high in extraversion had some unique navigational behavior patterns compared to those low in extraversion. For instance, they significantly navigated between viewing the course module and their personal achievements, where their grades, points and badges were displayed (CA1 → AR1). This could be because learners high in extraversion tend to be extrinsically motivated (Moldasheva and Mahmood, 2014), therefore they kept navigating to view their earned badges and points to motivate themselves. Furthermore, unlike learners low in extraversion, learners high in extraversion significantly navigated between seeing their peers’ profiles and the list of all peers (P2 → P1), meaning that they are not just looking up one person and then moving somewhere else, but seem to be looking up the profiles of multiple other learners. This could be explained with people high in extraversion are socializers and want to know new persons (Zhang, 2002; Tlili et al., 2019). Therefore, learners high in extraversion used the course as a place to build friendships by seeing the list of peers as well as their associated profiles.

As shown in Figure 3, learners low in extraversion, on the other hand, did not significantly focus on their personal achievements like their peers high in extraversion. However, they compared their achievements with that of their peers. Specifically, learners low in extraversion significantly navigated between their achievements and the list of peers, through which they could easily explore others’ profiles and achievements (AR1 ↔ P1 then P1 → P2 followed by P2 ↔ AR2). These navigational behavior patterns could be because learners low in extraversion are high achievement-driven (Mcclelland et al., 1953; Farley, 1966).

Based on the obtained findings, it is possible to enhance the course navigation experience for learners based on their extraversion personality. For instance, it is possible to make the rewards (e.g., points or badges) earned by learners within the course more visible on their profiles for those high in extraversion since they are more extrinsically motived. On the other hand, to motivate learners low in extraversion, it is better to make their progress more visible compared to their peers, for instance, through the use of leaderboard and progress bar. Denden et al. (2021) confirmed that compared to learners high in extraversion, learners low in extraversion found progress bar more useful.

### 4.2. Conscientiousness

The obtained chi-square test results confirmed that there is a significant relation between the rows and columns of the tallied frequencies (for high conscientiousness, *χ*^2^ = 7558.36, *df* = 121, *p* < 0.001; for low conscientiousness, *χ*^2^ = 5085.49, *df* = 121, *p* < 0.001). Figure 4 shows the navigational behavior patterns of learners high and low in conscientiousness.

As shown in Figure 4, unlike learners low in conscientiousness, those high in conscientiousness significantly navigated between viewing the course module and their achievements (CA1 → AR1). They then navigated to see their peers’ achievements (AR1 → P1; P1 → P2; P2 → AR2), where they kept checking their peers’ profiles and achievements (AR2 ↔ P2). This could be explained with learners high in conscientiousness are achievement-driven (Tlili et al., 2019), they therefore kept checking their course achievements and comparing them to their peers’ achievements. It is also found that learners high in conscientiousness significantly navigated to reading their peers’ discussions after seeing their achievements (AR2 → D1), but they were not involved in discussions (i.e., they did not write any comments, no D2). This could be explained with conscientious individuals are cautious about their public sayings in social networking websites, they therefore prefer sending private messages instead (Olson and Suls, 2000; Muscanell and Guadagno, 2012).

Interestingly, it is found that learners low in conscientiousness were also achievement-driven, however in a different way. Specifically, unlike learners high in conscientiousness, they mainly focused on their own achievements (AR1 → CA1) without going through others’ achievements (i.e., no AR2) (see Figure 4).

Based on the obtained findings, it is possible to enhance the course navigation experience for learners with conscientiousness personality. For instance, it is recommended that the developed online course should not mainly use public communication channels, such as forums, but also provide private ones, where learners high in conscientiousness will be more willing to involve in online social activities within the course. For example, software designers could develop a forum function that allows learners to write private messages to the persons involved in the discussion. On the other hand, it is recommended to develop some functionalities, such as badges and points, to make the course achievements for learners low in conscientiousness more visible, hence be more engaged while learning.

It should be noted that both learners high in conscientiousness and low in conscientiousness had unique navigational patterns (P2 → P1 and D1 → P2 for learners high in conscientiousness; P1 → AR1 for learners low in conscientiousness) that no explanation was found and further investigations need to be done to explain them.

### 4.3. Neuroticism

The chi-square test results revealed that the relationship between rows and columns of the tallied frequency is significant (*χ*^2^ = 4948.42, *df* = 121, *p* < 0.001 for learners high in neuroticism; *χ*^2^ = 7634.56, *df* = 121, *p* < 0.001 for learners low in neuroticism). Figure 5 shows the navigational behavior patterns of learners high and low in neuroticism.

As shown in Figure 5, unlike learners low in neuroticism, learners high in neuroticism went directly to see their associated personal achievements after they viewed the course/ module (CA1 → AR1). They also went to see the list of their peers and peers’ profiles where some of achievements had been displayed (P1 ↔ P2), and then went back to see their own achievements and compare (P1 → AR1). These two behavior sequences are explained with learners high in neuroticism have high level of anxiety and stress, and they always feel unsafe (Watjatrakul, 2016; Denden et al., 2021), and this was reflected in their behaviors where they significantly kept going back to see their course achievements and compared them to their peers, to know if they are doing well or not within the course. De Feyter et al. (2012) mentioned that neurotic learners cope with their anxiety about academic failure by intensifying their efforts in trying to prevent failure, which is seen in their online behavioral patterns of significantly checking their achievements and comparing them to their peers’ achievements.

Additionally, Figure 5 shows that, unlike learners low in neuroticism, learners high in neuroticism involved in discussions within the course forum (D2). Specifically, they significantly involved in discussions after they saw their assignment status, where teachers’ feedback and grades had been displayed (AS4 → D2) and they kept reading discussions (D2 → D1) and answering them (D2 ↔ D2). This could be explained with learners high in neuroticism used forums to communicate with their peers, as one of the ways to reduce their anxiety and stress, after seeing the feedback given by their teachers. Bhagat et al. (2019) also suggested that learners high in neuroticism should have the possibility to freely ask questions and talk with their peers to reduce their anxiety level and increase their chances of success.

On the other hand, Figure 5 shows that learners low in neuroticism, unlike learners high in neuroticism, significantly navigated between seeing their peers’ achievements and then specifically seeing their peers’ specific profiles (AR2 ↔ P2). This means that learners low in neuroticism visited the profiles of those they were interested in based their course achievements. Furthermore, they also went to the forum to view discussions possibly posted by their peers who they found interested in based on the course achievements (AR2 → D1). These behavioral sequences of keeping viewing peers’ profiles, achievements and discussions could be explained with persons low in neuroticism tend to be more self-confident and want to build connections within their environments (Baluku et al., 2016).

Moldasheva and Mahmood (2014) pointed out that learners high in neuroticism can get easily anxious. Therefore, to reduce their anxiety level within a given course and make them feel that they did well when learning online, it is possible to make the learning environment customizable where rewards, such as digital badges and points, can be configured to be visible for learners. Consequently, learners with high anxiety can see their course achievements and feel much better while learning. On the other hand, as learners low in neuroticism showed their willingness to discover people they were interested in, it would be helpful to develop and adopt more communication functionalities (i.e., public and private messaging) in the online learning environment, where learners are able to send messages privately to their peers to get to know them.

It should be noted that learners high in neuroticism had a unique navigational pattern, namely D1 → P1 that no explanation was found and further investigations need to be done to explain them.

### 4.4. Openness

The chi-square test results highlighted that there is a significant relationship between rows and columns of the behavior frequency tables of learners high (*χ*^2^ = 8527.14, *df* = 121, *p* < 0.001) or low (*χ*^2^ = 3556.29, *df* = 121, *p* < 0.001) in openness. Figure 6 shows the navigational behavior patterns of learners high and low in openness.

As shown in Figure 6, unlike those low in openness, learners high in openness conducted a lot of peers-related activities. Specifically, they were highly involved in discussions. For example, they engaged in discussions within the course forum after viewing their assignment status, where the grades and teachers’ feedback had been displayed (AS4 → D2). This behavior pattern could be explained by their willingness to know others’ opinions/experiences after reading the feedback given by the teacher. Furthermore, learners high in openness, unlike those low in openness, not only kept posting discussions (D2 → D2) but also continuously kept reading them (D2 → D1 and D1 → D1). These behavior sequences could possibly be the result of their inclination to exchange opinions with others. In this context, several studies pointed out that learners high in openness tend to be curious, imaginative, and creative. Moreover, they continuously seek out new experiences and are willing to take part in peer learning (Moldasheva and Mahmood, 2014; Tlili et al., 2019). The obtained findings about the discussion behavior patterns of learners high in openness are in line with previous research which indicates that openness is a significant predictor for the use of social networking sites (Banczyk et al., 2008; Krishnan and Atkin, 2014; Huang, 2019).

Furthermore, it is seen that learners high in openness checked their peers’ profiles and then saw their peers’ achievements following they viewed discussions (P2 ↔ AR2 and AR2 → D1). This is possibly because people high in openness are more likely to form a larger friendship network (Lang et al., 1998; Zhu et al., 2013). Specifically, they could consider the course forum as a place to meet new friends and further explored them by their profiles and achievements. Besides, it is interesting to see that after viewing peers’ achievement, learners high in openness could also go back to review the comments posted by the persons they were interested in (AR2 → D1).

Learners low in openness, on the other hand, were less interested in others’ experiences and mainly focused on their own experiences (see Figure 6). This can be seen when they significantly navigated between the course and their own achievements (CA1 → AR1). Interestingly, it can be seen that no significant behavioral pattern was found related to peers’ achievements (AR2) or discussion involvement (D1 or D2), unlike learners high in openness. This confirms that learners low in openness were not much interested in their peers’ experiences or achievements.

Based on the obtained findings, since learners high in openness are highly engaged in communication with others, it is recommended to provide them a learning environment which fosters various communication channels, such as forum or instant messages, to keep their learning engagement high. On the other hand, learners low in openness are less likely to get involved in discussions even after viewing their peers’ posts in the course forum, and they were more interested in their own course achievements. Therefore, it is recommended that learning environments should be designed with functionalities (e.g., dashboards or reports) that automatically generate the progress of learners in a given course to help them keep up with their progress and achievements.

## 5. Conclusions, implications and future directions

This study analyzed the impact of learners’ personality differences on their online learning navigation behavior patterns along four dimensions, namely: extraversion, conscientiousness, neuroticism, and openness. To the best of our knowledge, this study is one of the earliest studies that attempted to investigate how learners’ personalities might impact their navigational behavior patterns in an online course. The findings revealed that the learners’ extraversion level was found to affect their course navigation experience. Learners high in extraversion were more likely to have navigational behavior patterns, which were primarily influenced by extrinsic motivation. They navigated significantly between viewing peer profiles and all peer lists due to a greater desire to meet new people; conversely, learners low in extraversion navigated significantly between their achievements and peer lists due to their high achievement driven. The study also found that high levels of conscientiousness influenced learners’ online navigation behavior patterns due to being driven by achievement. Learners high in conscientiousness navigate significantly between viewing course modules and their achievements, and they tend to view their peers’ achievements and thus check their peers’ profiles and achievements; in contrast, learners low in conscientiousness were more likely to focus on their own achievements. Related to the neuroticism dimension, our study showed that learners with high neuroticism are usually accompanied by high levels of anxiety and stress, and they tend to look at their personal achievements directly after viewing the course/module, and they also compared their profiles and achievements with those of their peers; on the contrary, learners with low neuroticism are usually accompanied by self-confidence, and they tended to visit the profiles of people they are interested in based on their course grades. In addition, this present study showed that the level of openness also influences learners’ performance in peer-related activities. Those with high openness are highly engaged in discussions and learning and they expect to learn with their peers; conversely, those with low openness are focused on their own experiences and are not significant in terms of peer achievement and discussion participation.

This study can contribute to the literature in several ways. From a theoretical perspective, it can contribute to the educational psychology field by providing empirical evidences on how learners with different personalities tend to behave in an online course, hence better understand each personality trait and its related features in education. From a practical perspective, this study can contribute to the human-computer interaction and adaptivity fields by providing to various stakeholders (e.g., educators, designers, psychologists) several recommendations to enhance online course design and adaptivity with respect to each learner’s personality. It can also contribute to the learner modeling field by providing different behavioral patterns that could help to identify learners’ personalities. Finally, several studies have been conducted to automatically identify the learners’ personalities from their behaviors in an online course, these studies, however, focused on single behaviors, such as frequency of visiting a course or forum (Denden et al., 2018; Tlili et al., 2019). Therefore, the use of navigation behavioral patterns, from this present study, could be another data source for getting information about learners, hence making the automatic identification of their personalities more accurate.

It should be noted that this study has several limitations that should be acknowledged and further researched. For instance, the agreeableness personality trait was not investigated. Additionally, this study mainly investigated single personalities (e.g., learners high in openness vs. learners low in openness) and did not investigate combined personalities (e.g., learners high in openness and extraversion vs. learners low in openness and extraversion), which might reveal more information on learners’ online learning behaviors based on their personalities. Future research directions could focus on investigating these limitations, as well as designing an adaptive learning system based on personality, which takes into consideration the obtained findings of this study.

## Data availability statement

The raw data supporting the conclusions of this article will be made available by the authors, without undue reservation.

## Ethics statement

The studies involving human participants were reviewed and approved by The Smart Learning Institute of Beijing Normal University, China. Written informed consent for participation was not required for this study in accordance with the national legislation and the institutional requirements.

## Author contributions

All authors listed have made a substantial, direct, and intellectual contribution to the work and approved it for publication.

## Conflict of interest

The authors declare that the research was conducted in the absence of any commercial or financial relationships that could be construed as a potential conflict of interest.

## Publisher’s note

All claims expressed in this article are solely those of the authors and do not necessarily represent those of their affiliated organizations, or those of the publisher, the editors and the reviewers. Any product that may be evaluated in this article, or claim that may be made by its manufacturer, is not guaranteed or endorsed by the publisher.

## References

[ref1] Ackerman C. E. (2020). Big Fiver personality traits: the OCEAN model explained. Available at: https://positivepsychologyprogram.com/big- five-personality-theory/ (Accessed November 07, 2020).

[ref2] Al-Dujaily A. Kim J. Ryu H. (2013). Am I extravert or introvert? Considering the personality effect toward e-learning system. Educ. Technol. Soc. 16, 14–27.

[ref3] Arockiam L. Selvaraj J. C. (2013). User interface design for effective e-learning based on personality traits. Int. J. Comput. Appl. 61, 28–32. doi: 10.5120/9998-4861

[ref4] Bakeman R. Gottman J. M. (1997). Observing interaction: an introduction to sequential analysis. New York: Cambridge University Press.

[ref5] Bakeman R. Quera V. (1995). Analyzing interaction: sequential analysis with SDIS and GSEQ. UK: Cambridge University Press.

[ref6] Bakeman R. Quera V. (2011). Sequential analysis and observational methods for the behavioral sciences. Cambridge, UK: Cambridge University Press

[ref7] Baluku M. M. Kikooma J. F. Kibanja G. M. (2016). Does personality of owners of micro enterprises matter for the relationship between startup capital and entrepreneurial success? Afr. J. Bus. Manag. 10, 13–23. doi: 10.5897/AJBM2015.7738

[ref8] Banczyk B. Krämer N. C. Senokozlieva M. (2008). The wurst “meets, fatless” in MySpace. The relationship between personality, nationality and self- presentation in an online community. In Paper Presented at the Annual Conference of the International Communication Association, Montreal, Canada.

[ref9] Barrick M. Mount M. (1996). Effects of impression management and self-deception on the predictive validity of personality constructs. J. Appl. Psychol. 81, 261–272. doi: 10.1037/0021-9010.81.3.261, PMID: 8690688

[ref10] Barrick M. R. Mount M. K. Gupta R. (2003). Meta-analysis of the relationship between the five-factor model of personality and Holland's occupational types. Pers. Psychol. 56, 45–74. doi: 10.1111/j.1744-6570.2003.tb00143.x

[ref11] Bhagat K. K. Wu L. Y. Chang C. Y. (2019). The impact of personality on students' perceptions towards online learning. Australas. J. Educ. Technol. 35, 98–108. doi: 10.14742/ajet.4162, PMID: 36418787

[ref12] Bidjerano T. Yun Dai D. (2007). The relationship between the big-five model of personality and self-regulated learning strategies. Learn. Individ. Differ. 17, 69–81. doi: 10.1016/j.lindif.2007.02.001

[ref13] Boeree G. C. D. (2006). Personality theories. USA: Psychology Department Shippensburg University.

[ref14] Brusilovsky P. (2001). Adaptive hypermedia. User Model. User-Adap. Inter. 11, 87–110. doi: 10.1023/A:1011143116306, PMID: 36614587

[ref15] Chai H. Hu T. Niu G. (2022). How proactive personality promotes online learning performance? Mediating role of multidimensional learning engagement. Educ. Inf. Technol. 28, 4795–4817. doi: 10.1007/s10639-022-11319-7 PMC9589567 36311035

[ref16] Chen B. Resendes M. Chai C. S. Hong H. Y. (2017). Two tales of time: uncovering the significance of sequential patterns among contribution types in knowledge-building discourse. Interact. Learn. Environ. 25, 162–175. doi: 10.1080/10494820.2016.1276081

[ref17] Cheng H. N. Liu Z. Sun J. Liu S. Yang Z. (2017). Unfolding online learning behavioral patterns and their temporal changes of college students in SPOCs. Interact. Learn. Environ. 25, 176–188. doi: 10.1080/10494820.2016.1276082

[ref18] Cheng Y. Wang Y. Cheng I. Chen N. (2019). An in-depth analysis of the interaction transitions in a collaborative augmented reality-based mathematic game. Interact. Learn. Environ. 27, 782–796. doi: 10.1080/10494820.2019.1610448

[ref19] Child I. L. (1968). “Personality in culture” in Handbook of personality theory and research. eds. Borgatta E. Lambert W. W. (Chicago, IL: Rand McNally), 80–101.

[ref20] Clark M. H. Schroth C. A. (2010). Examining relationships between academic motivation and personality among college students. Learn. Individ. Differ. 20, 19–24. doi: 10.1016/j.lindif.2009.10.002, PMID: 36516637

[ref21] Codish D. Ravid G. (2014). Personality based gamification—educational gamification for extroverts and introverts. In Proceedings of the 9th Chais Conference for the Study of Innovation and Learning Technologies: Learning in the Technological Era. 36–44).

[ref22] Costa P. T. Jr McCrae R. R. (2009). “The Five-Factor Model and the NEO Inventories,” in Oxford handbook of personality assessment. Ed. J. N. Butcher (United States: Oxford University Press), 299–322.

[ref23] De Feyter T. Caers R. Vigna C. Berings D. (2012). Unraveling the impact of the Big Five personality traits on academic performance: The moderating and mediating effects of self-efficacy and academic motivation. Learn. Individ. Differ. 22, 439–448. doi: 10.1016/j.lindif.2012.03.013

[ref24] Denden M. Tlili A. Essalmi F. Jemni M. (2018). Implicit modeling of learners’ personalities in a game-based learning environment using their gaming behaviors. Smart Learn. Environ. 5, 1–19. doi: 10.1186/s40561-018-0078-6

[ref25] Denden M. Tlili A. Essalmi F. Jemni M. Chen N. Burgos D. (2021). Effects of gender and personality differences on students’ perceptions of game design elements in educational gamification. Int. J. Hum. Comput. Stud. 154:102674. doi: 10.1016/j.ijhcs.2021.102674

[ref26] DeYoung C. G. Quilty L. C. Peterson J. B. (2007). Between facets and domains: 10 aspects of the Big Five. J. Pers. Soc. Psychol. 93, 880–896. doi: 10.1037/0022-3514.93.5.880, PMID: 17983306

[ref27] Diseth A. (2003). Personality and approaches to learning as predictors of academic achievement. Eur. J. Personal. 17, 143–155. doi: 10.1002/per.469, PMID: 36761715

[ref28] Essalmi F. Tlili A. Ayed L. J. B. Jemni M. (2017). Toward modeling the learner's personality using educational games. Int. J. Distance Educ. Technol. 15, 21–38. doi: 10.4018/IJDET.2017100102, PMID: 34164437

[ref29] Farley F. H. (1966). Introversion and achievement motivation. Psychol. Rep. 19:112. doi: 10.2466/pr0.1966.19.1.112, PMID: 5942069

[ref30] Fatahi S. (2019). An experimental study on an adaptive e-learning environment based on learner’s personality and emotion. Educ. Inf. Technol. 24, 2225–2241. doi: 10.1007/s10639-019-09868-5

[ref31] Franić S. Borsboom D. Dolan C. V. Boomsma D. I. (2014). The Big Five personality traits: psychological entities or statistical constructs? Behav. Genet. 44, 591–604. doi: 10.1007/s10519-013-9625-7, PMID: 24162101

[ref32] Furnham A. (1992). Personality and learning style: a study of three instruments. Personal. Individ. Differ. 13, 429–438. doi: 10.1016/0191-8869(92)90071-V

[ref33] Graf S. Liu T. C. Kinshuk (2010). Analysis of learners' navigational behaviour and their learning styles in an online course. J. Comput. Assist. Learn. 26, 116–131. doi: 10.1111/j.1365-2729.2009.00336.x

[ref34] Gurven M. Rueden C. Massenkoff M. (2013). How universal is the Big Five? Testing the five-factor model of personality variation among forager-farmers in the Bolivian Amazon. J. Pers. Soc. Psychol. 104, 354–370. doi: 10.1037/a0030841, PMID: 23245291 PMC4104167

[ref35] Gustavsson J. P. Jönsson E. G. Linder J. Weinryb R. M. (2003). The HP5 inventory: definition and assessment of five health-relevant personality traits from a five-factor model perspective. Personal. Individ. Differ. 35, 69–89. doi: 10.1016/S0191-8869(02)00142-3

[ref36] Haron H. Sahar S. (2010). An investigation on predictors of e-learning adoption among Malaysian e-learners. In. International Conference on Science and Social Research (CSSR), 927–932.

[ref37] Harrington R. Loffredo D. A. (2010). MBTI personality type and other factors that relate to preference for online versus face-to-face instruction. Internet High. Educ. 13, 89–95. doi: 10.1016/j.iheduc.2009.11.006

[ref38] Hong J. Lee Y. Ye J. (2021). Procrastination predicts online self-regulated learning and online learning ineffectiveness during the coronavirus lockdown. Personal. Individ. Differ. 174:110673. doi: 10.1016/j.paid.2021.110673 PMC7846229 33551531

[ref39] Huang C. (2019). Social network site use and Big Five personality traits: a meta-analysis. Comput. Hum. Behav. 97, 280–290. doi: 10.1016/j.chb.2019.03.009, PMID: 26833246

[ref40] Irani T. Telg R. Scherler C. Harrington M. (2003). Personality type and its relationship to distance education students' course perceptions and performance. Q. Rev. Distance Educ. 4:445.

[ref41] John O. P. Robins R. W. Pervin L. A. (2008). Handbook of personality: theory and research. New York, NY: The Guilford Press.

[ref42] John O. P. Srivastava S. (1999). “The big-five trait taxonomy: history, measurement, and theoretical perspectives” in Handbook of personality: theory and research. eds. Pervin L. A. John O. P. (New York: Guilford Press), 102–138.

[ref43] Jonassen D. H. Grabowski B. L. (1993). “Handbook of individual differences” in Learning & Instruction (Hillsdale, NJ: Lawrence Erlbaum Associates)

[ref44] Krishnan A. Atkin D. (2014). Individual differences in social networking site users: the interplay between antecedents and consequential effect on level of activity. Comput. Hum. Behav. 40, 111–118. doi: 10.1016/j.chb.2014.07.045

[ref45] Kucuk S. Sisman B. (2017). Behavioral patterns of elementary students and teachers in one-to-one robotics instruction. Comput. Educ. 111, 31–43. doi: 10.1016/j.compedu.2017.04.002

[ref46] Lai S. Sun B. Wu F. Xiao R. (2019). Automatic personality identification using students’ online learning behavior. IEEE Trans. Learn. Technol. 13, 26–37. doi: 10.1109/TLT.2019.2924223

[ref47] Lang F. R. Staudinger U. M. Carstensen L. L. (1998). Perspectives on socioemotional selectivity in late life: How personality and social context do (and do not) make a difference. J. Gerontol. B 53B, 21–30. doi: 10.1093/geronb/53B.1.P21 9469168

[ref48] Lee J. M. Lee Y. (2006). Personality types and learners’ interaction in web-based threaded discussion. Q. Rev. Dist. Learn. 7, 83–94.

[ref49] Mcclelland D. C. Atkinson J. W. Clark R. A. Lowell E. L. (1953). The achievement motive. New York: Appleton-Century-Crofts.

[ref50] McComas J. Moore T. Dahl N. Hartman E. Hoch J. Symons F. (2009). Calculating contingencies in natural environments: issues in the application of sequential analysis. J. Appl. Behav. Anal. 42, 413–423. doi: 10.1901/jaba.2009.42-413, PMID: 19949534 PMC2695351

[ref51] McCrae R. R. John O. P. (1992). An introduction to the five-factor model and its applications. Personality 60, 175–215. doi: 10.1111/j.1467-6494.1992.tb00970.x 1635039

[ref52] Moldasheva G. Mahmood M. (2014). Personality, learning strategies, and academic performance: evidence from post-Soviet Kazakhstan. Educ. Train. 56, 343–359. doi: 10.1108/ET-10-2012-0101

[ref53] Murphy S. A. Fisher P. A. Robie C. (2021). International comparison of gender differences in the five-factor model of personality: an investigation across 105 countries. J. Res. Pers. 90:104047. doi: 10.1016/j.jrp.2020.104047

[ref54] Muscanell N. L. Guadagno R. E. (2012). Make new friends or keep the old: gender and personality differences in social networking use. Comput. Hum. Behav. 28, 107–112. doi: 10.1016/j.chb.2011.08.016, PMID: 37103713

[ref55] Myers I. B. McCaulley M. H. Quenk N. L. Hammer A. L. (1998). MBTI manual: a guide to the development and use of the Myers–Briggs type indicator (3). Palo Alto: Consulting Psychologists Press.

[ref56] Novikova I. A. Vorobyeva A. A. (2019). “The five-factor model: contemporary personality theory,” in Cross-cultural psychology: contemporary themes and perspectives. Ed. K. D. Keith, United States, 685–706.

[ref57] Olson B. D. Suls J. (2000). Self-, other-, and ideal-judgments of risk and caution as a function of the five-factor model of personality. Personal. Individ. Differ. 28, 425–436. doi: 10.1016/S0191-8869(99)00105-1

[ref58] Pohl M. Wallner G. Kriglstein S. (2016). Using lag-sequential analysis for understanding interaction sequences in visualizations. Int. J. Hum. Comput. Stud. 96, 54–66. doi: 10.1016/j.ijhcs.2016.07.006

[ref59] Sackett G. P. (1978). Observing behavior: I. Theory and applications in mental retardation. USA: University Park.

[ref60] Shang J. Xiao R. Zhang Y. (2020). A sequential analysis on the online learning behaviors of Chinese adult learners: take the KGC learning platform as an example. In International Conference on Blended Learning. 61–76). Thailand: Springer.

[ref61] Sun Z. Lin C. H. Lv K. Song J. (2021). Knowledge-construction behaviors in a mobile learning environment: a lag-sequential analysis of group differences. Educ. Technol. Res. Dev. 69, 533–551. doi: 10.1007/s11423-021-09938-x

[ref62] Sun T. Yu Q. Guo J. Ma Y. Zhang Y. (2020). A preliminary study of online learning: the influence of the class approaches and the personality of students. Advances in Social Science, Education and Humanities Research, 496, 382–391.

[ref63] Swanberg A. B. Martinsen L. (2010). Personality, approaches to learning and achievement. Educ. Psychol. 30, 75–88. doi: 10.1080/01443410903410474

[ref64] Tlili A. Denden M. Essalmi F. Jemni M. Chang M. Kinshuk. (2019). Automatic modeling learner’s personality using learning analytics approach in an intelligent Moodle learning platform. Interact. Learn. Environ. doi: 10.1080/10494820.2019.1636084

[ref65] Tlili A. Essalmi F. Jemni M. Chen N. S. (2016). Role of personality in computer based learning. Comput. Hum. Behav. 64, 805–813. doi: 10.1016/j.chb.2016.07.043, PMID: 37084915

[ref66] Tlili A. Wang H. Gao B. Shi Y. Zhiying N. Looi C. K.. (2021). Impact of cultural diversity on students’ learning behavioral patterns in open and online courses: A lag sequential analysis approach. Interact. Learn. Environ. 1–20. doi: 10.1080/10494820.2021.1946565

[ref67] Trull T. J. Sher K. J. (1994). Relationship between the five-factor model of personality and Axis I disorders in a nonclinical sample. J. Abnorm. Psychol. 103:350. doi: 10.1037/0021-843X.103.2.350, PMID: 8040504

[ref68] Wampold B. (1992). “The intensive examination of social interactions” in Single-Case Research Design and Analysis: New Directions for Psychology and Education. eds. Kratochwill T. R. Levin J. R. (Hillsdale, NJ: Lawrence Erlbaum Associates), 93–131.

[ref69] Wang F. H. (2017). An exploration of online behavioral engagement and achievement in flipped classroom supported by learning management system. Comput. Educ. 114, 79–91. doi: 10.1016/j.compedu.2017.06.012

[ref70] Wang C. Shannon D. M. Ross M. E. (2013). Students’ characteristics, self-regulated learning, technology self-efficacy, and course outcomes in online learning. Distance Educ. 34, 302–323. doi: 10.1080/01587919.2013.835779

[ref71] Wang H. Tlili A. Lämsä J. Cai Z. Zhong X. Huang R. (2022). Temporal perspective on the gender-related differences in online learning behaviour. Behav. Inform. Technol. 1–15. doi: 10.1080/0144929X.2022.2039769

[ref72] Watjatrakul B. (2016). Online learning adoption: effects of neuroticism, openness to experience, and perceived values. Interact. Technol. Smart Educ. 13, 229–243. doi: 10.1108/ITSE-06-2016-0017

[ref73] Wengrowicz N. Swart W. Paul R. Macleod K. Dori D. Dori Y. J. (2018). Students’ collaborative learning attitudes and their satisfaction with online collaborative case-based courses. Am. J. Dist. Educ. 32, 283–300. doi: 10.1080/08923647.2018.1511509

[ref74] Wu S. Hou H. (2015). How cognitive styles affect the learning behaviors of online problem-solving based discussion activity: A lag sequential analysis. J. Educ. Comput. 52, 277–298. doi: 10.1177/0735633115571307

[ref75] Yang X. Li J. Guo X. Li X. (2015). Group interactive network and behavioral patterns in online English-to-Chinese cooperative translation activity. Internet High. Educ. 25, 28–36. doi: 10.1016/j.iheduc.2014.12.003

[ref76] Yang X. Wang H. Li J. (2016). The application of lag sequential analysis method in analyzing learning behavior. China Educ. Technol. 2, 17–23.

[ref77] Yu Z. (2021). The effects of gender, educational level, and personality on online learning outcomes during the COVID-19 pandemic. Int. J. Educ. Technol. High. Educ. 18:14. doi: 10.1186/s41239-021-00252-3 34778520 PMC8016506

[ref78] Zhang L. (2002). Thinking styles and the big five personality traits. Educ. Psychol. 22, 17–31. doi: 10.1080/01443410120101224, PMID: 36044535

[ref79] Zhang L. (2003). Does the big five predict learning approaches? Personal. Individ. Differ. 34, 1431–1446. doi: 10.1016/S0191-8869(02)00125-3, PMID: 37007551

[ref80] Zhang S. Liu Q. Chen W. Wang Q. Huang Z. (2017). Interactive networks and social knowledge construction behavioral patterns in primary school teachers’ online collaborative learning activities. Comput. Educ. 104, 1–17. doi: 10.1016/j.compedu.2016.10.011

[ref81] Zhu X. Woo S. E. Porter C. Brzezinski M. (2013). Pathways to happiness: from personality to social networks and perceived support. Soc. Networks 35, 382–393. doi: 10.1016/j.socnet.2013.04.005, PMID: 28374005

