# Supplementary material for: Impact of personality traits on learners’ navigational behavior patterns in an online course: a lag sequential analysis approach
Source: Front Psychol. 2023 May 23;14:1071985. doi: 10.3389/fpsyg.2023.1071985 (PMC10245556; doi:10.3389/fpsyg.2023.1071985)
Supplement: Supplementary file 1 [file Data_Sheet_1.pdf]

Appendix. Behavior frequency distribution based on the four personality dimensions

Behavior	Personality	Frequency	Z	Sig.
CA1	LowExtra	30.93	-.816	.415
	HighExtra	34.77		
	LowCon	30.29	-1.208	.227
	HighCon	35.97		
	LowNeuro	35.72	-1.215	.224
	HighNeuro	30.02		
	LowOp	28.90	-1.619	.105
	HighOp	36.51		
CA2	LowExtra	32.17	-.331	.740
	HighExtra	33.71		
	LowCon	32.82	-.079	.937
	HighCon	33.19		
	LowNeuro	30.37	-1.184	.237
	HighNeuro	35.89		
	LowOp	28.58	-1.756	.079
	HighOp	36.79		
AS1	LowExtra	37.63	-1.848	.065
	HighExtra	29.03		
	LowCon	32.12	-.398	.691
	HighCon	33.97		
	LowNeuro	33.88	-.398	.691
	HighNeuro	32.03		
	LowOp	28.35	-1.854	.064
	HighOp	36.99		
AS2	LowExtra	36.65	-1.479	.139
	HighExtra	29.87		
	LowCon	31.69	-.600	.549
	HighCon	34.44		
	LowNeuro	32.60	-.182	.856
	HighNeuro	33.44		
	LowOp	30.08	-1.182	.237
	HighOp	35.50		
AS3	LowExtra	37.23	-1.903	.057
	HighExtra	29.37		
	LowCon	30.47	-1.286	.198
	HighCon	35.77		
	LowNeuro	35.72	-1.384	.166
	HighNeuro	30.02		
	LowOp	31.30	-.764	.445
	HighOp	34.46		
AS4	LowExtra	33.83	-.329	.742
	HighExtra	32.29		

	LowCon	31.96	-.467	.641
	HighCon	34.15		
	LowNeuro	33.22	-.099	.921
	HighNeuro	32.76		
	LowOp	27.32	-2.246	.025
	HighOp	37.87		
D1	LowExtra	32.82	-.135	.893
	HighExtra	33.16		
	LowCon	31.35	-1.367	.172
	HighCon	34.81		
	LowNeuro	33.35	-.293	.770
	HighNeuro	32.61		
	LowOp	31.53	-1.076	.282
	HighOp	34.26		
D2	LowExtra	33.65	-.706	.480
	HighExtra	32.44		
	LowCon	32.47	-.650	.516
	HighCon	33.58		
	LowNeuro	32.43	-.704	.481
	HighNeuro	33.63		
	LowOp	31.50	-1.629	.103
	HighOp	34.29		
AR1	LowExtra	36.10	-1.236	.217
	HighExtra	30.34		
	LowCon	31.37	-.736	.462
	HighCon	34.79		
	LowNeuro	34.56	-.703	.482
	HighNeuro	31.29		
	LowOp	27.10	-2.351	.019
	HighOp	38.06		
AR2	LowExtra	32.62	-.416	.677
	HighExtra	33.33		
	LowCon	31.50	-1.842	.065
	HighCon	34.65		
	LowNeuro	34.37	-1.680	.093
	HighNeuro	31.50		
	LowOp	32.57	-.471	.638
	HighOp	33.37		
P1	LowExtra	31.42	-.708	.479
	HighExtra	34.36		
	LowCon	27.19	-2.939	.003
	HighCon	39.37		
	LowNeuro	36.15	-1.592	.111
	HighNeuro	29.55		
	LowOp	29.25	-1.677	.094
	HighOp	36.21		
P2	LowExtra	28.95	-1.666	.096
	HighExtra	36.47		

	LowNeuro	36.32	-1.547	.122
	HighNeuro	29.35		
	LowNeuro	36.32	-1.547	.122
	HighNeuro	29.35		
	LowOp	27.73	-2.167	.030
	HighOp	37.51		
